# Composite materials with enhanced dimensionless Young’s modulus and desired Poisson’s ratio

**DOI:** 10.1038/srep14103

**Published:** 2015-09-11

**Authors:** H. X. Zhu, T. X. Fan, D. Zhang

**Affiliations:** 1 School of Engineering, Cardiff University, Cardiff, CF24 3AA, UK; 2 State Key Lab of Metal Matrix Composites, Shanghai Jiaotong University, Shanghai, 200240, China

## Abstract

We have designed a new type of composite materials which not only has a Young’s modulus much larger than the Voigt limit, but also is always nearly isotropic. Moreover, its Poisson’s ratio can be designed at a desired value, e.g. positive, or negative, or zero. We have also demonstrated that structural hierarchy can help to enhance the stiffness of this type of composite materials. The results obtained in this paper provide a very useful insight into the development of new functional materials and structures.

Our life quality and living conditions largely rely on composite materials. In fact, the bones in our body are a nano-structured hierarchical composite material with the basic building blocks being nano-sized single crystal mineral plates embedded in soft protein matrix^1^,^2^. Many different types of advanced artificial composite materials are used more and more frequently in our daily lives, examples include kitchen tools, sport facilities, vehicle and airplane structures.

The Voigt limit has long been regarded as an unexceedable upper limit for the stiffness of isotropic composite materials, as can be seen from thousands of text books, e.g. reference^3^. For a two phase composite made of two different isotropic materials A and B whose Young’s moduli are *E*_*A*_ and *E*_*B*_, and Poisson ratios are *v*_*A*_ and *v*_*B*_, respectively, the Voigt limit for the Young’s modulus of the composite is given as


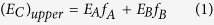


Where *f*_*A*_ and *f*_*B*_ are the volume fractions of the two materials, and thus *f*_*A*_ + *f*_*B*_ = 1. The lower limit (i.e. the Reuss limit) for the Young’s modulus of the two-phase composite is given as


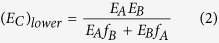


It is relatively easier to make the Young’s modulus of an anisotropic composite equal to or larger than the Voigt limit than an isotropic composite. For example, for a laminate composite made of two isotropic materials, the in-plane Young’s modulus is obviously the same as the Voigt limit if the Poisson’s ratios of the two component materials are the same, and larger than the Voigt limit if the Poisson’s ratios are different. The larger the difference of the two Poisson’s ratios, the larger the stiffness of the laminate composite. In general, laminate composites may have 3 orthogonal planes of symmetry, thus they may have up to 9 independent elastic constants. If a laminate composite is in-plane isotropic, the number of the independent elastic constants will reduce to 5 from 9.

Lim^4^ has investigated the out-of-plane modulus of semi-auxetic laminates, and found that out-of-plane stiffness can be made larger than the Voigt limit by using a combination of positive and negative Poisson’s ratios. Liu *et al.*^5^ have analyzed the elastic properties of in-plane isotropic semi-auxetic laminates and obtained all the 5 independent elastic constants. They found that both the in-plane and out-of-plane moduli can be made larger than the Voigt limit using a combination of positive and negative Poisson’s ratios. Lim and Rajendra Acharya^6^ and Grima *et al.*^7^ have also studied the elastic properties of semi-auxetic laminates.

As semi-auxetic laminate composites are in general orthotropic, they may have 5 or a larger number of independent elastic constants. The objective of this paper is to design a new type of composite materials which not only has a Young’s modulus much larger than the Voigt limit, but more importantly, is always nearly isotropic. The Poisson’s ratio can be designed at a desired value, e.g. positive, or negative, or zero, and structural hierarchy can further enhance the Young’s modulus.

## Geometric and Mechanics Model

**Figure 1:**
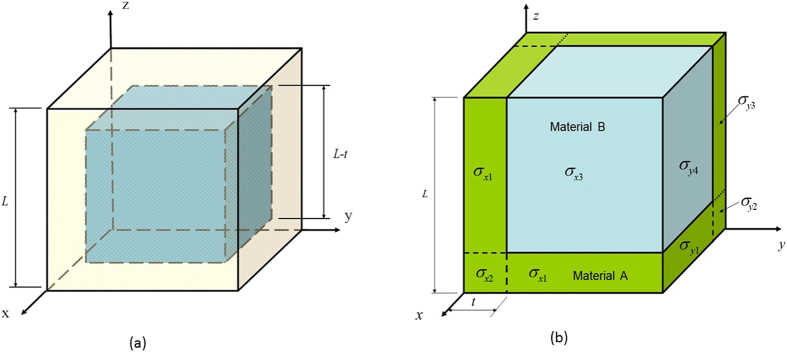
A cubic periodic representative volume element (RVE) of the two-phase composite material. (**a**) A cubic periodic unit RVE (**b**) Cubic periodic mechanics model.

The emphasis of this paper is on the design of single-level two-phase composite materials. The designed single-level composite materials are assumed to be composed of a large number of identical cubic periodic cells, as shown in Fig. 1(a) which is one representative volume element (RVE) of the composite. In the RVE, material A is a hollow cubic box which has square walls of uniform thickness *t/*2 and an external edge length *L*; material B is a solid cube which is inside the hollow cubic box of material A and has an edge length *L – t.* The interfaces of materials A and B are assumed to be perfectly bonded. Advanced manufacturing technology, e.g. 3D printing or prototyping^8^, makes it possible to produce such designed composite material.

In the two-phase composite, the volume fraction of material A is


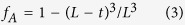


and the volume fraction of material B is thus 
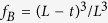
.

The designed composite material has a cubic symmetry and thus has only up to 3 independent elastic constants^9^,^10^, namely *E*_*xx*_, *G*_*xy*_ and *v*_*xy*_. Obviously, *E*_*yy*_ = *E*_*zz*_ = *E*_*xx*_, *G*_*xz*_ = *G*_*yz*_ = *G*_*xy*_ and *v*_*yz*_ = *v*_*xz*_ = *v*_*xy*_, and the Zener’s anisotropy factor is always very close to 1 (i.e. nearly isotropic). To obtain the effective Young’s modulus *E*_*xx*_ and the Poisson’s ratio *v*_*xy*_ for the composite material, the cubic periodic RVE shown in Fig. 1(a) is stretched to a strain *ε*_*x*_ in the *x* direction by an effective uniaxial tensile force/stress. The periodic boundary conditions and the symmetry of the applied load require that all the six outside planes of the cubic periodic unit RVE in Fig. 1(a) remain plane after deformation.

To simplify the analysis, the RVE is divided into 8 parallelepipeds, as can be seen in Fig. 1(b). In order to carry out analytical solution, we consider only the normal stresses within each of the 8 parallelepipeds in the RVE and the periodic conditions (i.e. compatibility conditions) on the outside surfaces of the RVE, and ignore the shear stresses inside the parallelepipeds and the compatibility conditions on the interfaces between the parallelepipeds inside the RVE. Thus, the cubic periodic representative volume element (RVE) shown in Fig. 1(b) can be used as a simplified mechanics model of the two-phase composite, where the 3 normal stresses in each of the 8 parallelepipeds are assumed to have constant values. When the RVE is stretched in the *x* direction, the normal stresses and strains on the top plane of the RVE shown in Fig. 1(b) are exactly the same as those on the right plane. According to the symmetry, we have 7 different unknown normal stresses, namely, σ_*x*1_, σ_*x*2_ and σ_*x*3_ on the front surface of the RVE; and σ_*y*1_, σ_*y*2_, σ_*y*3_ and σ_*y*4_ on the right surface of the RVE, as shown in Fig. 1(b). From the Hooke’s law and the periodic boundary conditions of the RVE, we have following stress-strain relations


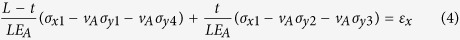



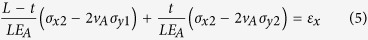



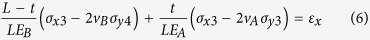



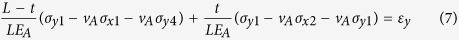



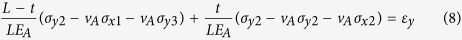



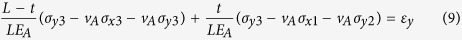



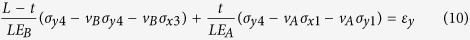


In addition, the zero total force in the normal direction of the top or right plane of the RVE in Fig. 1(b) requires


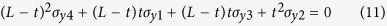


For a given value of the tensile strain *ε*_*x*_, we have in total only 8 unknowns to be determined: σ_*x*1_, σ_*x*2_, σ_*x*3_, σ_*y*1_, σ_*y*2_, σ_*y*3_, σ_*y*4_, and *ε*_*y*_. They can be solved from the 8 simultaneous linear Equations (4, 5, 6, 7, 8, 9, 10, 11). Thus, the effective Young’s modulus and Poisson’s ratio of the composite material can be obtained as


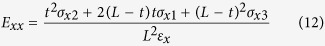



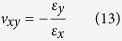


## Results

It is well known that the range of the Poisson’s ratio of isotropic materials is from −1.0 to 0.5, i.e. 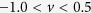 (refs 11, 12, 13, 14, 15, 16). For example, solid polymer or rubber materials, or low density random irregular open cell foams^12^ have a Poisson’s ratio close to 0.5; most metal materials have a Poisson’s ratio between 0.1 and 0.4; cork has a Poisson’s ratio close to 0 (ref. 17); open cell foams with re-entrant cells (i.e. auxetic foams) have a negative Poisson’s ratio^13^,^14^; hierarchical laminates^18^ or auxetic materials^19^ can be designed to be isotropic and to have a Poisson’s ratio close to −1.0 (refs 13, 14, 15, 16,19).

**Figure 2:**
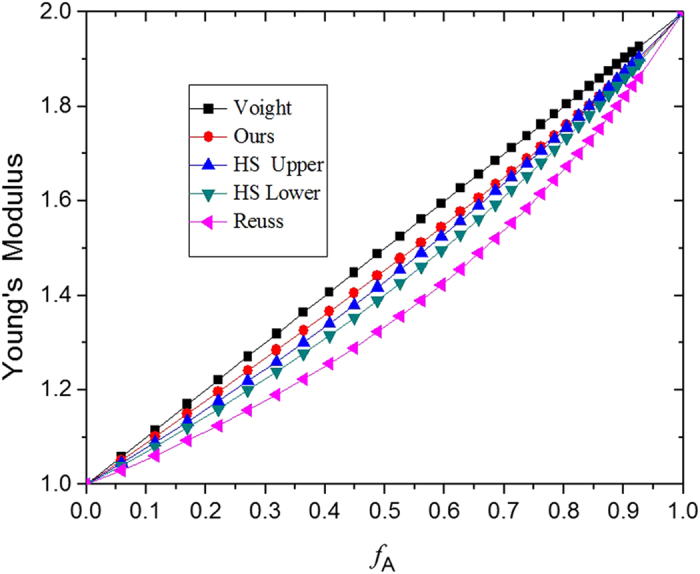
Young’s modulus of the two-phase composite with the cubic periodic RVE structure shown in Fig. 1 **and with** ***E***_***A***_** = 2.0*****E***_***B***_ **and** ***v***_***A***_** = *****v***_***B***_ **vs. the volume fraction of material A, compared with the Voigt limit, the Reuss Limit, and the Hashin-Shtrikman upper and lower limits.** The Young’s moduli are normalized by *E*_*B*_ in Fig. 2.

For single-level two-phase composite materials with the cubic periodic RVE structure shown in Fig. 1 and with *E*_*A*_ = 2.0*E*_*B*_ and 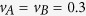, the relationship between the effective Young’s modulus *E*_*xx*_ and the volume fraction *f*_*A*_ can be obtained by solving Equations 3, 5, 6, 7, 8, 9, 10, 11, 12 and plotted in Fig. 2. The Voigt bound, the Reuss bound, and the Hashin—Shtrikman^20^ upper and lower bounds are also presented for comparison. It is noted that the Young’s moduli in Fig. 2 are normalized by *E*_*B*_. As can be seen from Fig. 2, the effective Young’s modulus of the composite material predicted from our mechanics model shown in Fig. 1(b) is larger than the Hashin—Shtrikman upper limit when the volume fraction *f*_*A*_ is smaller than 82%. As the possible effect of the Poisson’s ratios of materials A and B is completely absent in Fig. 2, the enhancement of the effective Young’s modulus (i.e. larger than the Hashin—Shtrikman upper limit) can be attributed to the geometrical structure. We have also tested cases of 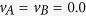 and other values, and found that as long as 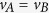, the results of the effective Young’s modulus of the composite materials obtained from Equations (3, 4, 5, 6, 7, 8, 9, 10, 11, 12) remain unchanged.

**Figure 3:**
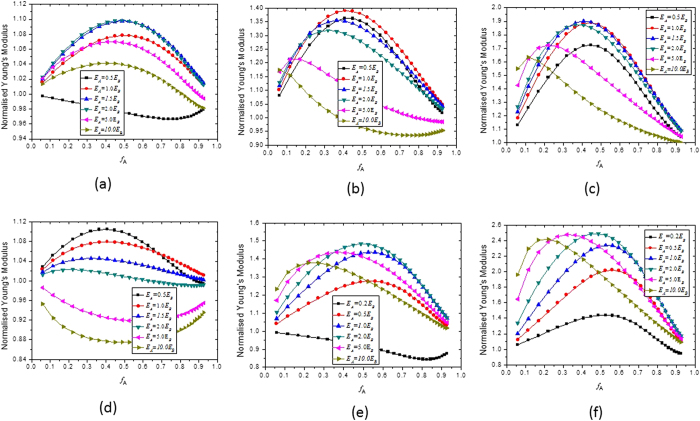
Effects of the value of *E*_*A*_/*E*_*B*_ on the relationship between the normalized Young’s modulus of the composites and the volume fraction of material A: (**a**) *v*_*A*_ = 0.05 and *v*_*B*_ = 0.495; (**b**) *v*_*A*_ = 0.45 and *v*_*B*_ = −0.5; (**c**) *v*_*A*_ = 0.45 and *v*_*B*_ = −0.8; (**d**) *v*_*A*_ = 0.495 and *v*_*B*_ = 0.05; (**e**) *v*_*A*_ = −0.5 and *v*_*B*_ = 0.45; (**f**) *v*_*A*_ = −0.8 and *v*_*B*_ = 0.45.

We now explore how to make the Young’s modulus of a single-level composite material larger than the Voigt limit. For a single-level two-phase composite material with the cubic periodic RVE structure shown in Fig. 1, the effects of different combinations of the Young’s moduli and Poisson’s ratios of materials A and B on the relationship between the effective Young’s modulus of the composite and the volume fraction *f*_*A*_ are illustrated in Fig. 3(a–f), where the Young’s modulus of the composite is normalized by the Voigt limit 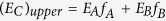. As the Voigt limit normalized by itself is constantly 1.0, a value above 1.0 in Fig. 3(a–f) indicates that the Young’s modulus of the composite material is larger than the Voigt limit.

We can see from Fig. 3(a–f) that, when *E*_*A*_ = *E*_*B*_ (i.e. when the possible effects of the difference between *E*_*A*_ and *E*_*B*_ are absent), the difference between *v*_*A*_ and *v*_*B*_ can make the normalised Young’s modulus of the composite material larger than 1.0 (i.e. exceeding the Voigt limit). Moreover, the larger the difference between *v*_*A*_ and *v*_*B*_, the larger the Young’s modulus of the two-phase composite material. Comparing Fig. 3(b,c) to (e,f), it can be found that if *v*_*A*_ is negative and *v*_*B*_ is positive, the composite material has a larger Young’s modulus than the case when *v*_*A*_ is positive and *v*_*B*_ is negative. In the case when *v*_*A*_ = −0.8 and *v*_*B*_ = 0.45, the Young’s modulus of the composite material is about 150% larger than the Voigt limit.

**Figure 4:**
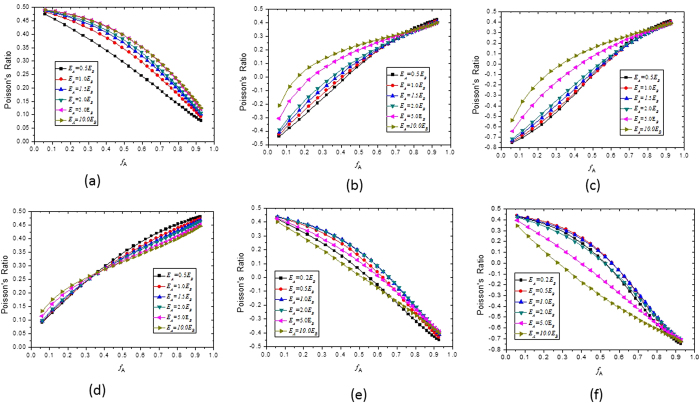
Effects of the value of *E*_*A*_/*E*_*B*_ on the relationship between the Poisson’s ratio of the composite and the volume fraction of material A: (**a**) *v*_*A*_ = 0.05, *v*_*B*_ = 0.495; (**b**) *v*_*A*_ = 0.45, *v*_*B*_ = −0.5; (**c**) *v*_*A*_ = 0.45, *v*_*B*_ = −0.8; (**d**) *v*_*A*_ = 0.495, *v*_*B*_ = 0.05; (**e**) *v*_*A*_ = −0.5 and *v*_*B*_ = 0.45; (**f**) *v*_*A*_ = −0.8 and *v*_*B*_ = 0.45.

Figure 4(a–f) show that by properly choosing the Young’s moduli and the Poisson’s ratios of materials A and B, the Poisson’s ratio of a two-phase composite material can be designed to have a desired value, e.g. positive, or negative, or zero. These results are very useful for the design of more interesting and useful functional materials or structures for applications in many different areas. For example, materials with a zero Poisson’s ratio are perfect for sealing applications^17^.

## Discussion

To validate the analytical results for the effective Young’s moduli and Poisson’s ratios of the two-phase composite materials obtained from Equations (4, 5, 6, 7, 8, 9, 10, 11), we used the commercial finite element software ABAQUS to perform a number of simulations (i.e. to do numerical experiments) for the cubic periodic RVE structural model shown in Fig. 1(a). The RVE is partitioned into 8000 C3D8 elements. Periodic boundary conditions are used in all the finite element simulations and the obtained simulation results can be assumed to be the exact results. Table 1 presents the analytical results and the finite element simulation results for the two-phase composite materials with different combinations among the values of *E*_*A*_, *E*_*B*_, *v*_*A*_, *v*_*B*_ and *f*_*A*_, where the effective Young’s moduli of the composites are normalized by the Voigt limit (*E*_*C*_)_*upper*_.

Table 1 shows that the analytical results for the Young’s modulus of the single-level composite materials obtained from Equations (4, 5, 6, 7, 8, 9, 10, 11) are always smaller than the simulation results, suggesting that the analytical results always tend to underestimate the Young’s modulus of the composite materials. This is consistent with the mechanics principle because any additional restraint always makes a material or structure stiffer. In the analysis of Equations (4, 5, 6, 7, 8, 9, 10, 11), only normal stresses in the RVE and periodic conditions on the outside boundaries of the RVE are considered, while all the possible shear stresses and all the compatibility conditions inside the RVE are ignored. This could result in possible gaps or overlaps between the 8 deformed parallelepipeds inside the RVE. To remove the gaps and overlaps (i.e. to make the interfaces between the 8 deformed parallelepipeds inside the RVE perfectly bonded), additional work has to be done, and this consequently increases the stored strain energy in the RVE and hence makes the composite stiffer. In contrast, all the actual normal and shear stresses and all the compatibility conditions inside and outside the RVE have already been considered in the finite element simulations using the ABAQUS software. As the finite element simulations have considered much more restraints between the interfaces of the 8 parallelepipeds than the simplified mechanics model shown in Fig. 1(b), the exact results for the effective Young’s modulus obtained from the finite element simulations are consequently always larger than the analytical results obtained from Equations (4, 5, 6, 7, 8, 9, 10, 11).

Table 1 shows that when *v*_*B*_ ≥ −0.8, the difference between the effective Young’s modulus of the composite materials obtained from Equations (4, 5, 6, 7, 8, 9, 10, 11) and that obtained from the ABAQUS finite element simulation is constantly less than 8%, indicating that the analytical results shown in Figs 3 and 4 are quite accurate and hence reliable. When *v*_*B*_ approaches −1.0, although the error of the analytical results becomes larger, the predicted trend of the effects remains correct.

**Figure 5:**
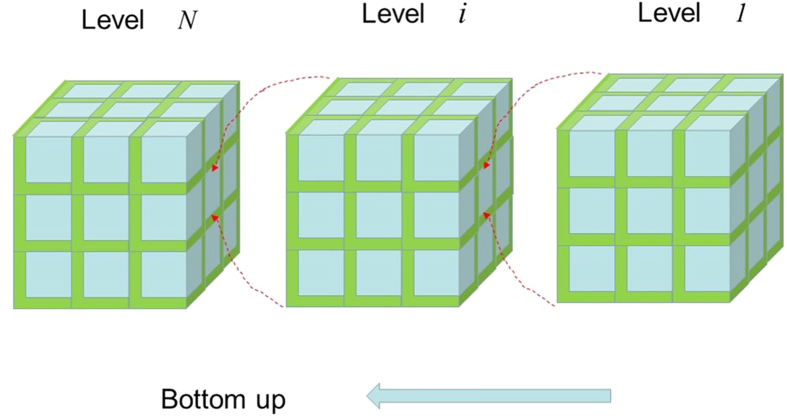
Bottom-up structure of hierarchical composites.

Now we demonstrate how structure hierarchy could further enhance the elastic properties of a two-phase composite material. The two-phase hierarchical composite material is assumed to be made of isotropic materials A and B with Young’s moduli *E*_*A*_ and *E*_*B*_, Poisson ratios *v*_*A*_ and *v*_*B*_, and volume fraction *f*_*B*_. At each hierarchical level *n*, the composite material is assumed to be composed of a large number of identical RVEs, as shown in Fig. 5, and each of the cubic fillers/inclusions (i.e. equivalent to material ‘B’ in Fig. 1) in the RVEs is also made of a large number of identical lower level (i.e. level *n* − 1) cubic periodic RVEs. For simplicity, the hierarchical composite material is assumed to be self-similar in structure, and the volume fraction of the cubic fillers/inclusions (i.e. material ‘B’ ) in the RVEs is assumed to remain fixed at all hierarchical levels^21^,


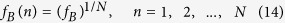


Where, *n* is the specific hierarchical level and *N* is the total number of the hierarchical levels.

**Figure 6:**
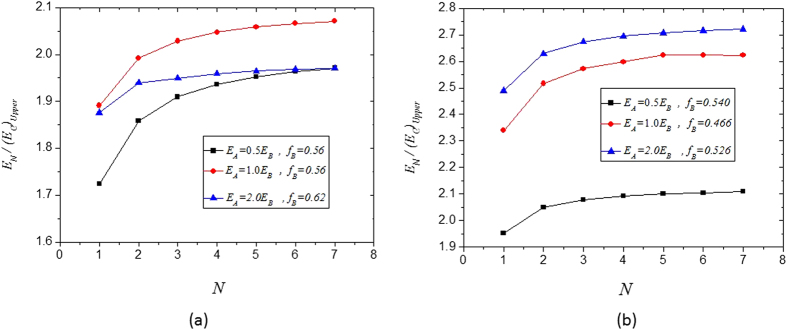
Dimensionless Young’s modulus of hierarchical composites as a function of the total number of the hierarchical levels: (**a**) *v*_*A*_ = 0.45, *v*_*B*_ = −0.8; (**b**) *v*_*A*_ = −0.8 and *v*_*B*_ = 0.45.

**Figure 7:**
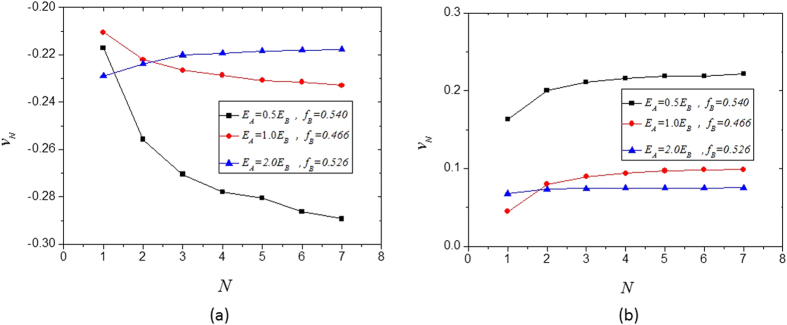
Poisson’s ratio of hierarchical composites as a function of the total number of the hierarchical levels: (**a**) *v*_*A*_ = 0.45, *v*_*B*_ = −0.8; (**b**) *v*_*A*_ = −0.8 and *v*_*B*_ = 0.45.

For a given material volume fraction *f*_*B*_ and a given number of the total hierarchical levels *N*, the volume fraction of the cubic fillers/inclusions in the RVEs at each hierarchical level, *f*_*B*(*n*)_, can be obtained from Equation (14), and the Young’s modulus *E*_(*n*)_ and Poisson’s ratio *v*_(*n*)_ at each hierarchical level can be obtained from Equations (4, 5, 6, 7, 8, 9, 10, 11, 12, 13). Figures 6 and 7 show the analytical results of the Young’s modulus *E*_*N*_ and Poisson ratio *v*_*N*_ for a few hierarchical and self-similar composite materials as functions of the number of the total hierarchical levels *N*, where the Young’s modulus is normalized by the Voigt limit 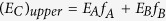. In Figs 6 and 7, the results of the case *N* = 1 are those of the single-level composites, which can also be seen from Figs 3(c,f) and 4(c,f). The results in Fig. 6(a,b) indicate that increasing the number of hierarchical levels tends to enhance the stiffness of composite materials. The results obtained in this paper provide very useful insight into the development of new functional materials and structures.

## Additional Information

**How to cite this article**: Zhu, H.X. *et al.* Composite materials with enhanced dimensionless Young’s modulus and desired Poisson’s ratio. *Sci. Rep.* **5**, 14103; doi: 10.1038/srep14103 (2015).

## Figures and Tables

**Table 1:** Comparison between the analytical results and finite element simulation results.

Composite material	**Analytical results**		**Simulation results**	
	***E*_*xx*_/(*E*_*C*_)_*upper*_**	***v*_*xy*_**	***E*_*xx*_/(*E*_*C*_)_*upper*_**	***v*_*xy*_**
*E*_*A*_ = 2*E*_*B*_ *f*_*A*_ = 0.271 *v*_*A*_ = 0.05 *v*_*B*_ = 0.495	1.0794	0.4453	1.0858	0.4550
*E*_*A*_ = 2*E*_*B*_ *f*_*A*_ = 0.271 *v*_*A*_ = 0.45 *v*_*B*_ = −0.8	1.8128	−0.3981	1.8930	−0.3792
*E*_*A*_ = 2*E*_*B*_ * f*_*A*_ = 0.271 *v*_*A*_ = 0.495 *v*_*B*_ = −0.99	2.9665	−0.8896	3.5342	−0.9175
*E*_*A*_ = 2*E*_*B*_ *f*_*A*_ = 0.488 *v*_*A*_ = 0.05 *v*_*B*_ = 0.495	1.0986	0.3828	1.1156	0.3920
*E*_*A*_ = 2*E*_*B*_ *f*_*A*_ = 0.488 *v*_*A*_ = 0.45 *v*_*B*_ = −0.8	1.8216	−0.0680	1.9637	−0.0237
*E*_*A*_ = 2*E*_*B*_ *f*_*A*_ = 0.488 *v*_*A*_ = 0.495 * v*_*B*_ = −0.9	2.5586	−0.2554	2.9617	−0.1282
*E*_*A*_ = 2*E*_*B*_ *f*_*A*_ = 0.488 *v*_*A*_ = 0.495 *v*_*B*_ = −0.95	3.0174	−0.4095	3.9841	−0.2998
*E*_*A*_ = 2*E*_*B*_ * f*_*A*_ = 0.488 *v*_*A*_ = 0.495 *v*_*B*_ = −0.99	3.6526	−0.4976	5.5141	−0.5817
